# Spontaneous Subcapsular Renal Hematoma as Cause of Hypovolemic Shock in a Chronic Hemodialysis Patient

**DOI:** 10.1155/2023/5570992

**Published:** 2023-09-11

**Authors:** Jorge Luis Bermudez-Gonzalez, Bethsabel Rodríguez-Encinas, Ángel Miguel Beverido-Florido, Jesús Alejandro Gabutti-Thomas

**Affiliations:** ^1^Graduate Studies Division, Medicine School, National Autonomous University of Mexico, Mexico City, Mexico; ^2^Education Department, National Institute of Medical Sciences and Nutrition Salvador Zubiran, Mexico City, Mexico; ^3^Interventional Radiology Department, National Institute of Medical Sciences and Nutrition Salvador Zubiran, Mexico City, Mexico

## Abstract

Subcapsular renal hematomas may appear spontaneously in patients in chronic hemodialysis, though other causes as neoplasms, vasculitis, and infections should be excluded. Patients may present with abdominal pain and hemorrhagic shock; hence, early diagnosis is vital. Nephrectomy or renal artery embolization are suitable treatment options.

## 1. Introduction

Subcapsular renal hematomas (SRH) are rare and usually trauma-related [1]; however, spontaneous presentation occurs mainly in patients with malignancies, vasculitis, infection, or hemodialysis [2, 3]. Traditionally, spontaneous SRH present as abdominal pain, hematuria, and other signs of bleeding [4]. Herein, we report the case of a nontrauma-related spontaneous SRH in a patient in chronic hemodialysis which presented as hemorrhagic shock in the emergency department and was successfully managed with renal artery embolization.

## 2. Case Presentation

A 53-year-old man presented to the emergency department complaining of excruciating abdominal pain of 2 hours of onset while he was lying in bed and watching TV. He self-administered acetaminophen without resolution of the symptoms, with pain being the chief complaint for his emergency department consultation. He had a medical history of diabetes, hypertension, peripheral artery disease, end-stage chronic kidney disease on hemodialysis, and several emergency department visits for diabetic foot which concluded in a supracondylar amputation of his left leg one month before. His current medications were linagliptin, sevelamer, erythropoietin, aspirin, atorvastatin, omeprazole, amlodipine, pregabalin, duloxetine, and buprenorphine.

His vital signs at admission showed hypotension and tachycardia. Physical examination was remarkable for severe abdominal pain at palpation of the right superior quadrant, with no visible signs like bruises or petechiae. He also had retarded capillary filling. His laboratory tests showed a decreased hemoglobin level of 7.6 g/dL (previous of 9.8 g/dL), leukocytosis (19 × 10^3^/*μ*L), and an elevated C-reactive protein (3.4 mg/dL). A peripheral venous access was established, and crystalloids were administered.

**Figure 1:**
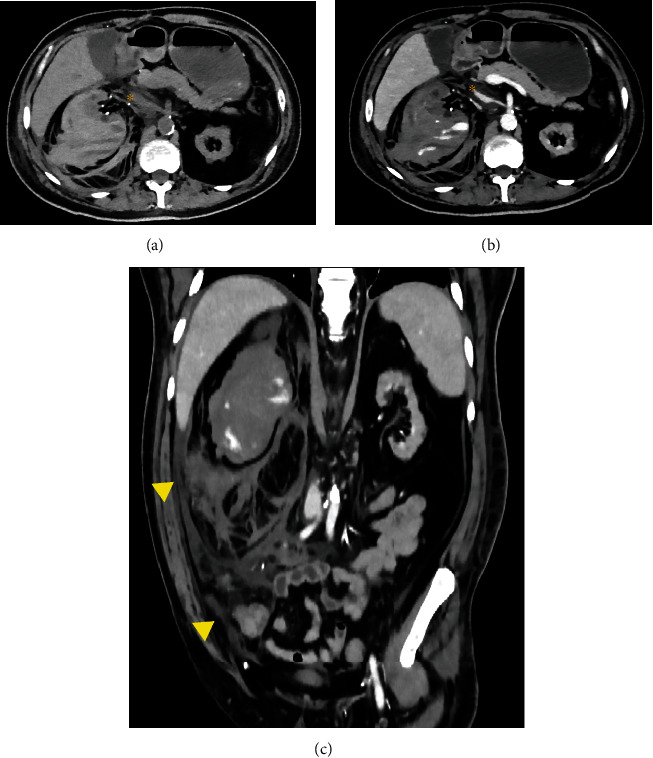
Axial nonenhancement (a) and venous phase CT (b) show increased right kidney size secondary to subcapsular and perirenal hematoma conditioning inferior vena cava displacement (^∗^). Coronal venous phase CT (c) shows hematic fluid along the right lateral paracolic gutter to the ipsilateral iliac fossa (arrowhead).

**Figure 2:**
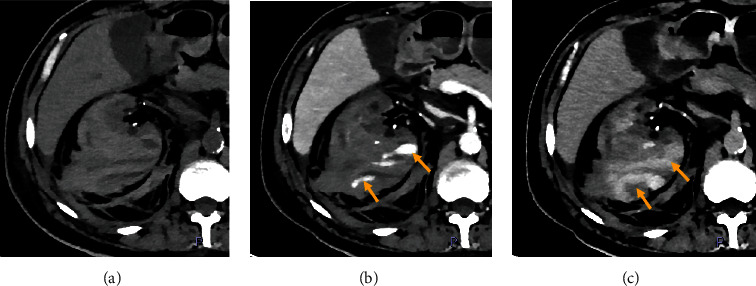
Axial nonenhancement (a), portal venous (b), and delayed phases (c) CT images show a subcapsular and perirenal hematoma on the right kidney. The portal venous phase (b) shows contrast extravasation (arrows) and changes in size, attenuation, and shape in the delayed phase (c).

A CT angiography was performed due to suspicion of abdominal bleeding. The study showed a subcapsular and perirenal hematoma in the right kidney (Figure 1). Signs of active bleeding as contrast medium extravasation were noticed in the inferior renal pole (Figure 2). The CT images also showed a decreased caliber of the inferior vena cava (due to hypovolemia and external compression) and anterior displacement of the head of the pancreas and duodenum. The left kidney was atrophic with cysts.

**Figure 3:**
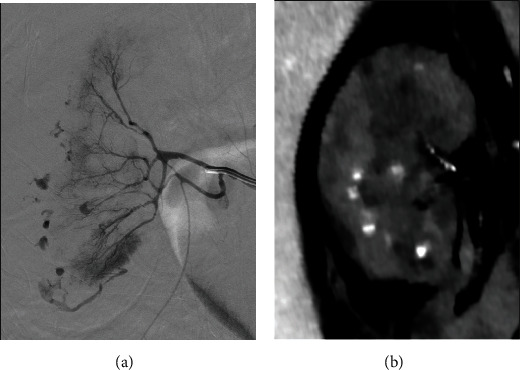
Digital subtraction angiography of the right renal artery (a) and coronal venous phase CT image (b) demonstrate active extravasation from the subsegmental arteries.

**Figure 4:**
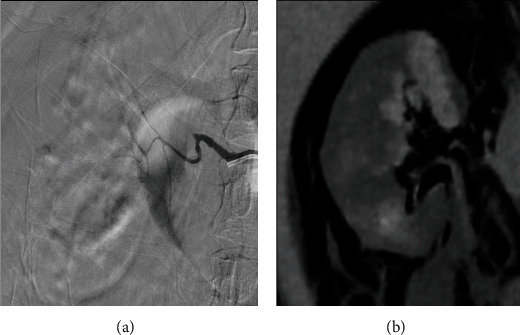
Digital subtraction angiography of the right renal artery (a) demonstrates embolization of the bleeding vessel with no residual bleeding. Coronal venous phase CT image (b) shows a decrease in the size of the subcapsular hematoma and absence of bleeding foci.

Urgent urology and interventional radiology consultations were requested. New blood tests showed further decrease in hemoglobin (5.5 g/dL), and two units of packed red blood cells were infused. The patient was transferred to the angiography room where subtraction renal angiography demonstrated at least twelve sites of active arterial bleeding **(**Figure 3). Due to these findings, renal artery embolization was performed using PVA microparticles (250-355 microns). Fifteen minutes later, a new angiography showed the absence of right renal artery flux and no signs of further bleeding (Figure 4). The patient was transferred to the intensive care unit for vigilance, where he did not bleed again, and 4 days later he was discharged home.

## 3. Discussion

First reported by Bonet and later described by Wünderlich, SRH (also referred as Wünderlich's syndrome when there are non-traumatic causes) is defined as a parenchymal bleeding confined to the subcapsular space of the kidney, with hemoperitoneum being rare giving the self-limiting bleeding due to the resistance of the perirenal tissue. [4, 5].

Spontaneous SRH may present as abdominal pain, a palpable mass, and signs of internal bleeding such as hypovolemic shock or hematuria (Lenk's triad). With some patients presenting with hemorrhagic shock, early diagnosis and management is crucial, but the clinical presentation varies broadly. Spontaneous SRH is usually preceded by urinary tract manipulation, such as catheterization or kidney biopsy, and has also been described as an early complication during kidney transplantation and as a cause of graft injury and failure [6].

The main etiologies reported are tumors, with malignant tumors such as adenocarcinoma, followed by angiomyolipoma, being the most common. Angiomyolipoma can be found either isolated or associated with tuberous sclerosis, where they usually present at younger ages, reaching larger sizes, multifocal, and bilateral [7, 8].

Literature describing this condition is generally limited to case series or case reports. The bigger series describes kidney neoplasms as the most common cause of spontaneous SRH, followed by vasculitis, infections, and idiopathic [1]. However these series may not truly represent the current epidemiology of the twenty-first century, and recent studies are lacking. In a recent report by Singh et al. [9], they describe the etiology of six recently published cases of spontaneous SRH. Three of them were caused by urinary tract manipulation, one by anticoagulation, other by antiplatelet therapy, and only one by an invasive mole. In addition, according to a 2019 series of cases in Mexico of at least around 53 patients with SRH, this entity presented in more than 60% of the cases in the female sex, with underlying pathologies reported such as pyelonephritis, cancer, angiomyolipomas, and renal cysts [10]. There is also a high prevalence of comorbidities such as diabetes mellitus and systemic arterial hypertension in our population.

Other proposed risk factors that have been described are anticoagulation, antiplatelet drugs, non-steroidal anti-inflammatory drugs, hypertension, and blood dyscrasia [3, 5].

Hemorrhagic events are common among chronic hemodialysis patients [11]. The pathophysiology has not been elucidated, but it has been theorized that severe atherosclerosis, anticoagulation during hemodialysis, high blood pressure and concurrent illnesses may play a vital role in creating a prohemorrhagic environment. Otherwise, it could appear counterintuitive that even hypothrophic and nonfunctional kidneys could be an important source of bleeding [12].

In the setting of current bleeding, nephrectomy and renal artery embolization are the main treatment options. For stable patients, conservative management has been proposed as a suitable option. In older series, nephrectomy is recommended above all options due to the high frequency of malignancies found among excised kidneys [2]. However, in scenarios of hemodynamic instability such as our patient, active management is preferred [3]. To our knowledge, spontaneous SRH is a rare entity and appears to be more common among chronic hemodialysis patients even without anticoagulation. In conclusion, despite being a rare condition, with still little known in our local epidemiology, it is a life-threatening condition and should be recognized promptly so it can be managed appropriately.

## Data Availability

All data underlying this case report are available as part of the article, and no additional source data are required.
